# Autonomic function and motor subtypes in Parkinson’s disease: a multicentre cross-sectional study

**DOI:** 10.1038/s41598-023-41662-9

**Published:** 2023-09-04

**Authors:** Si-Chun Gu, Rong Shi, Chen Gao, Xiao-Lei Yuan, You Wu, Zhen-Guo Liu, Chang-De Wang, Shao-Rong Zhao, Xiqun Chen, Can-Xing Yuan, Qing Ye

**Affiliations:** 1 Department of Neurology, Longhua Hospital, Shanghai University of Traditional Chinese Medicine, 725 South Wanping Road, Shanghai, 200032 China; 2 Department of Emergency, Shuguang Hospital, Shanghai University of Traditional Chinese Medicine, 528 Zhangheng Road, Shanghai, 201203 China; 3 Department of Neurology, Xinhua Hospital, Shanghai Jiao Tong University School of Medicine, 1665 Kongjiang Road, Shanghai, 200092 China; 4 Department of Neurology, Shanghai TCM-Integrated Hospital, Shanghai University of Traditional Chinese Medicine, 230 Baoding Road, Shanghai, 200082 China; 5 Department of Neurology, Putuo District Central Hospital, Shanghai University of Traditional Chinese Medicine, 164 Lanxi Road, Shanghai, 200062 China; 6 Department of Neurology, Massachusetts General Hospital, Harvard Medical School, 114 16th Street, Charlestown, MA 02129 USA

**Keywords:** Neuroscience, Motivation, Motor control, Neural ageing, Peripheral nervous system, Neurological disorders, Movement disorders, Neurodegenerative diseases, Parkinson's disease, Peripheral neuropathies

## Abstract

Autonomic symptoms (AS) are critical in Parkinson’s disease (PD). We aimed to determine the relative significance of clinical factors allowing predictions about incidence of AS, and examine AS profiles among PD patients by motor subtype and its relation to AS. The cross-sectional data of a multicentre sample, including 714 PD patients and 194 healthy controls from Parkinson’s Progression Marker Initiative study and Pingchan granule study were analyzed, stratified by PD subtypes [postural instability and gait disturbances (PIGD), tremor dominant (TD), and indeterminate] and domain autonomic dysfunction. Compared with healthy controls, PD patients scored higher in the total Scales for Outcomes in Parkinson's Disease-Autonomic dysfunction score and in several domain scores in particular, and there was a significant overlap in domain AS. Risk factors of individual domain autonomic dysfunction were heterogeneous. PIGD and indeterminate were the predominant subtypes in pupillomotor and thermoregulatory symptoms. TD and indeterminate were more likely to suffer from cardiovascular problem. The odd in sexual dysfunction was significant for PIGD. Gastrointestinal and urinary symptoms seemed not to be associated with a specific subtype. Our study demonstrated that AS were highly heterogeneous and 3 subtypes differed in autonomic performance, providing clues to understand mechanisms underlying AS in PD.

## Introduction

Parkinson’s disease (PD) is one of the most common neurodegenerative disorders and characterized by its insidious onset and progressive motor symptoms of tremor, bradykinesia, rigidity and postural instability^1^. It has increasingly been acknowledged that the clinical spectrum of PD is more extensive covering various non-motor symptoms (NMS), such as autonomic symptoms (AS), neuropsychiatric disturbances, sleep disorders or cognitive impairment as common clinical features^2,3^. AS in PD are critical and present many debilitating manifestations such as urinary and pupillomotor problems, constipation, cold or heat intolerance, orthostatic light-headedness, swallowing and sweating problems because of the involvement of multiple autonomic nervous system (ANS) domains. Moreover, AS might occur in all PD patients virtually at some stage of their course and contribute to the disease burden^4–6^. Therefore the systematic assessment and identification of AS in PD is of great value in clinical practice for avoiding complications and requiring appropriate treatment.

Currently, PD can be subclassified into three motor subtypes: postural instability and gait disturbance (PIGD), tremor dominant (TD), and indeterminate^7^. Increasing evidence showed that there were significant links between NMS and clinical motor subtypes, and NMS seemed to vary according to motor subtypes^8–10^. In respect to cognitive impairment, PIGD was associated with greater cognitive decline such as executive functions, attention and memory compared with TD^8^. TD scored lower in cognitive tests such as digit span, word fluency and attention as compared with indeterminate. As for sleep disorders, PIGD underwent more severe excessive daytime sleepiness (EDS) and PD-related sleep problems (PD-SP)^9^. Furthermore, it was reported that PIGD had higher sensitivity for depression compared to other subtypes^10^.

In spite of the recent increase of interest in AS, many aspects of AS in PD remain open questions. Main limitation of previous relating research on AS lied in the fact that they concentrated on a specific domain of the ANS and (or) on a single autonomic symptom^11–13^. In addition, inconsistencies existed among previous studies, in particular with respect to the contributory factors of AS such as age, duration and other NMS^14,15^. Furthermore, what is concerning is that the relationship between AS and the clinical motor subtypes in PD has not been discovered, which might hinder the individualised diagnosis and therapy interventions of AS in the higher risk group particularly at the earliest stage of disease. Thus, there is a growing need for a detailed investigation of AS in PD.

In this study, we aimed to identify distinct clinical characteristics with respect to the motor subtype (PIGD, TD, and indeterminate) in the entire group of patients with PD as well as within each domain autonomic dysfunction group (patients with gastrointestinal, urinary, cardiovascular, thermoregulatory, pupillomotor, and sexual symptoms), which could help identify a baseline AS profile and allow predictions about incidence of six domain autonomic dysfunction, as well as their possible associations to motor subtypes.

## Methods

### Study design

All methods were performed in accordance with the relevant guidelines and regulations. We conducted a cross-sectional study embedded within the Pingchan granule (PCG) study and the Parkinson’s Progression Marker Initiative (PPMI) study in accordance with The Strengthening the Reporting of Observational Studies in Epidemiology (STROBE) Statement (see Supplementary Material 1). The outcome measures for the PCG study and the PPMI study were identical and baseline data from both studies were extracted. The PCG study was a randomized, double-blind, placebo-controlled trial conducted in 4 hospitals in China aiming to evaluate the efficacy and safety of PCG for motor and non-motor symptoms of PD^16^. The PCG trial has been registered in Chinese Clinical Trial Register, number ChiCTR-INR-17011949 (http://www.chictr.org.cn/showproj.aspx?proj=20043) and followed the Consolidated Standards of Reporting Trials Extension (CONSORT Extension) reporting guideline strictly with the approval of ethics committee of Longhua Hospital with the following ethic code: 2017LCSY326, and subsequently by the relevant ethics committees at all sites (see Supplementary Material 2). In this trial, eligible participants were enrolled and randomly allocated at a 1:1 ratio to PCG or placebo groups. A sample size of 292 trial population with 146 participants per arm was required with consideration of attrition rate. Randomization was performed by random permuted blocks of sizes four to provide a balanced distribution of treatment groups. PCG and placebo were allocated to participants by interviewers, which were identical and could not be differentiated. Blind methods were applied for both participants and researchers including interviewers and assessors of the outcomes. PPMI is a large, multicentre, prospective population-based cohort that follows PD patients and healthy controls (HC) in 33 sites for identifying PD progression biomarkers (www.ppmi-info.org)^17^. And each participating site in PPMI study received approval from an ethical standard committee on human experimentation and obtained written informed consent (https://www.ppmi-info.org/about-ppmi/ppmi-clinical-site). For up-to-date information on the study, visit www.ppmi-info.org.

### Study population

Inclusion criteria for analysis were a diagnosis of PD according to the Movement Disorder Society (MDS) Clinical Diagnostic Criteria, age over 30, and Hoehn and Yahr stage 1–3 at baseline^18,19^. Exclusion criteria were receiving treatments for psychiatric disorders; any significant medical conditions (other than PD) that could impede full participation in the study; use of reserpine, metoclopramide, α-methyldopa, amphetamine derivatives, or methylphenidate within the past 3 months; participating in other clinical trials, and the women who were pregnant or lactating. Use of levodopa and concomitant anti-parkinsonian medications such as anticholinergic drugs, MAO-B inhibitors, amantadine, catechol-O-methyltransferase inhibitors, or dopamine agonists was allowed if dosages were stable for at least 30 days before enrollment. All HC were recruited from PPMI cohort. They were not on any long-term drug therapy, had no significant medical history, no first degree family member with PD and Montreal Cognitive Assessment (MOCA) > 26^20^.

### Clinical evaluation

All the PD patients in PPMI study and PCG study completed the Movement Disorder Society Unified Parkinson’s Disease Rating Scale (MDS-UPDRS)^21^. The PD motor subtypes were defined using the MDS-UPDRS parts II and III, including the tremor dominant (TD) subtype (ratio ≥ 1.5), postural instability and gait disturbance (PIGD) subtype (ratio ≤ 1) and indeterminate subtype (ratios > 1.0 and < 1.5)^22^. The presence of AS was evaluated with the Scales for Outcomes in Parkinson's Disease-Autonomic dysfunction (SCOPA-AUT) questionnaire. The SCOPA-AUT is divided into 6 domains and contains questions addressing gastrointestinal, urinary, cardiovascular, thermoregulatory, pupillomotor, and sexual symptoms^23^. Excessive daytime sleepiness (EDS) was defined as the Japanese version of the Epworth Sleepiness Scale (ESS) score of 10 or greater^24^. Rapid Eye Movement (REM) sleep behavior disorder (RBD) symptoms were assessed by the Japanese version of the RBD screening questionnaire (RBDSQ-J). And probable RBD (pRBD) was defined as an RBDSQ-J score of 5 or greater^25^. Depressive symptoms were evaluated with the 15-item Geriatric Depression Scale (GDS-15), and a GDS-15 score of 5 or greater was defined as clinically significant depressive disturbances in PD^26^. Cognitive impairment was estimated by the MOCA scale. The levodopa equivalent dose (LED) was calculated based on previously reported conversion factors^27^.

### Statistical analysis

The baseline data of PPMI study and PCG study were analyzed. The Shapiro–Wilk statistic was applied for testing the normality of the distribution of data. Continuous data were presented as mean (SD) or median (interquartile range [IQR]), with categorical data presented as proportion and number where appropriate. For categorical data, comparisons were analyzed by Chi-square tests or Fisher’s exact tests. For continuous data, comparisons were analyzed by Student’s t-tests or Wilcoxon’s rank-sum tests.

Considering the differences in the baseline characteristics between PD patients and HC, propensity score matching (PSM) was applied for identifying a cohort with similar baseline characteristics^28^. We applied a nonparsimonious multivariable logistic-regression model, with group as the dependent variable and age, education as covariates to evaluate the propensity score. Matching was conducted following a 1:1 protocol without replacement, with a caliper width equal to 0.05 of the standard deviation of the logit of the propensity score. Standardized differences before and after matching were calculated for all covariates to assess prematch and postmatch balance. And standardized differences of less than 10.00% indicated a relatively small imbalance. PSM was performed with MatchIt package^29^.

Multivariable stepwise logistic regression models were applied to screen risk factors of domain autonomic dysfunction, with age, age of onset, sex, education, PD duration, Hoehn and Yahr stage, MDS-UPDRS I, II, III and IV scores, EDS, pRBD, depression, MOCA, LED, and clinical motor subtypes used as the independent variables. Akaike’s information criterion (AIC) was used as the selection criteria in each final model. For sensitivity analyses, we also applied gradient boosting regression tree (GBDT) to rank the importance of variables with respect to their correlation of AS. GBDT is an ensemble machine learning algorithm combining weak ‘learners’ into a strong single learner in an iteration fashion. When comparing SCOPA-AUT domain and total scores among subtypes, differences were adjusted by variables in logistic regression and GBDT models^30^.

Differences between mean total SCOPA-AUT scores, in relation to stage of PD, were explored with use of the Kruskal–Wallis H test. In addition, the correlation between total SCOPA-AUT score and stage of PD was also measured using the Spearman’s rho correlation coefficient. Two-tailed *p* values of less than or equal 0.05 were considered statistically significant. All statistical analyses were performed with R software (version 3.3.3).

## Results

292 PD patients from PCG study (PCG group: n = 146, placebo group: n = 146), as well as 422 PD patients and 194 HC from PPMI cohort were included (Table 1). Only 2.64% of subjects had 0 total SCOPA-AUT score, showing that there was not a test floor effect. A ceiling effect was also not demonstrated since the highest observed total SCOPA-AUT score was 41 (1 PD patient) while only 0.84% of PD patients scored over 30. As for HC, the highest SCOPA-AUT total score was 20 (1 control). Before PSM, there were differences in age and education of the baseline between patients and controls. To solve the imbalance and potential bias, PSM was used and 186 PD patients were matched with 186 controls (Table 1). The standardized differences after PSM were less than 10.00%, indicating there were only small differences between the two groups. After matching, PD patients scored higher than controls in the total SCOPA-AUT score in general and in the gastrointestinal, urinary, cardiovascular, and sexual dysfunction domain scores in particular, with the urinary domain score having the highest level.

**Table 1:** Baseline characteristics before and after propensity score matching.

Characteristics	Before matching				After matching			
	Controls (n = 194)	PD (n = 714)	Standardized difference	*p* Value	Controls (n = 186)	PD (n = 186)	Standardized difference	*p* Value
Age, years (median [IQR])	62.46 [55.44, 68.97]	65.00 [59.00, 70.00]	0.296	0.001	62.83 [55.95, 69.11]	62.89 [56.02, 68.48]	0.027	0.897
Gender female, n (%)	69 (35.56)	276 (38.66)	0.064	0.482	66 (35.5)	57 (30.65)	0.103	0.378
Education, years (median [IQR])	16.00 [14.00, 18.00]	14.00 [11.00, 16.00]	0.644	< 0.001	16.00 [14.00, 18.00]	16.00 [14.00, 18.00]	0.042	0.811
SCOPA-AUT total score (median [IQR])	5.83 (3.69)	9.18 (6.28)	0.650	< 0.001	5.94 (3.70)	8.69 (5.50)	0.587	< 0.001
Gastrointestinal domain (mean (SD))	0.66 (1.01)	2.65 (2.46)	1.059	< 0.001	0.68 (1.02)	2.24 (2.29)	0.878	< 0.001
Urinary domain (mean (SD))	3.06 (2.14)	3.76 (2.98)	0.271	0.002	3.11 (2.14)	3.75 (2.77)	0.257	0.014
Cardiovascular domain (mean (SD))	0.19 (0.45)	0.57 (0.95)	0.515	< 0.001	0.18 (0.45)	0.46 (0.82)	0.412	< 0.001
Thermoregulatory domain (mean (SD))	0.86 (1.07)	1.00 (1.59)	0.108	0.108	0.88 (1.06)	0.73 (1.28)	0.120	0.249
Pupillomotor domain (mean (SD))	0.29 (0.52)	0.22 (0.53)	0.136	0.095	0.30 (0.53)	0.19 (0.53)	0.205	0.051
Sexual function domain (mean (SD))	0.78 (1.30)	0.66 (1.31)	0.090	0.268	0.78 (1.27)	0.83 (1.33)	0.120	0.033

Abbreviations: *PD* Parkinson’s Disease, *SCOPA-AUT* Scales for Outcomes in Parkinson’s Disease–Autonomic Dysfunction, *IQR* interquartile range.

**Figure 1:**
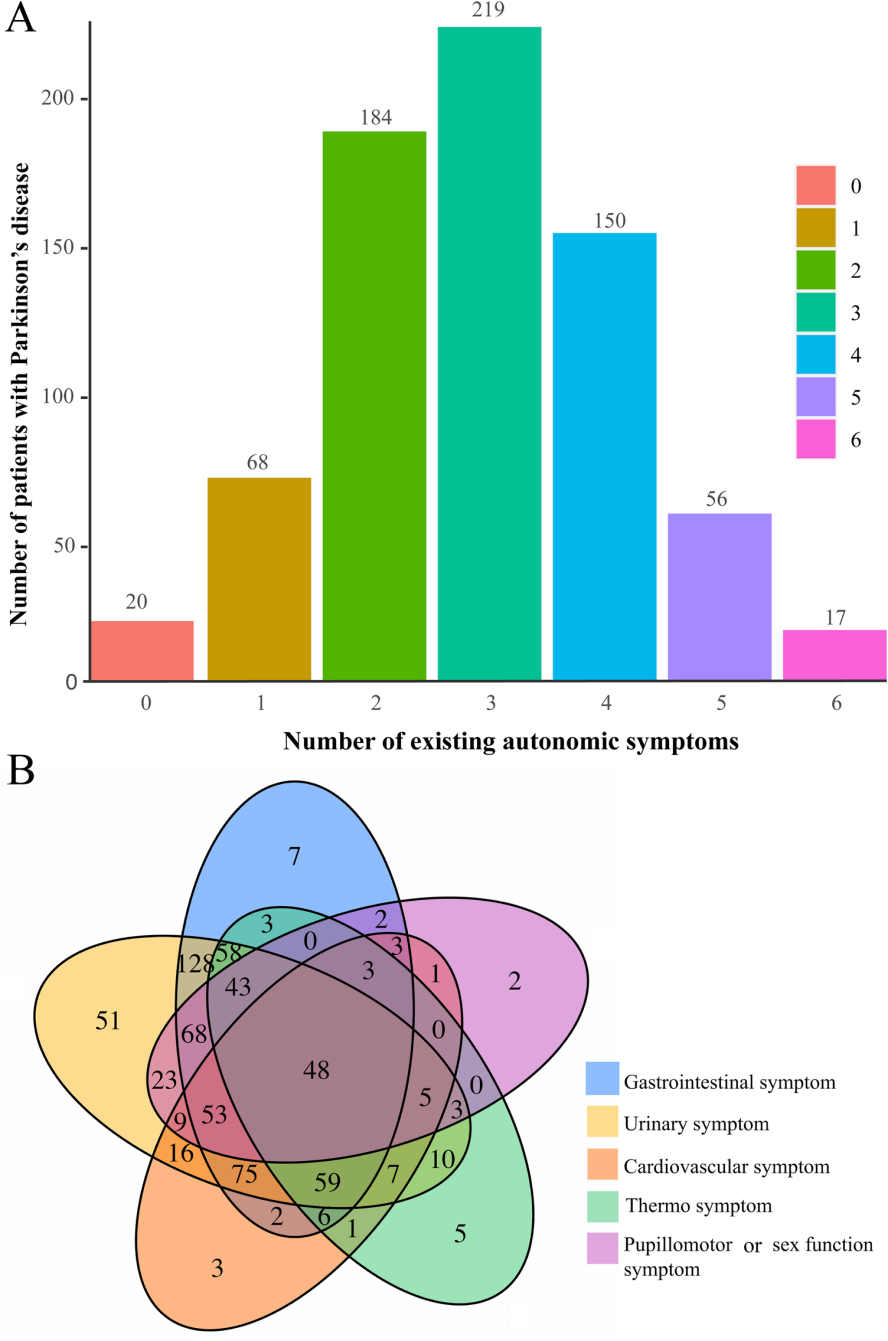
(**A**) Prevalence of coexisting autonomic symptoms in patients with PD. (**B**) Prevalence and overlap of autonomic symptoms in patients with PD. PD, Parkinson’s disease. All figures were created with the use of R software (version 3.3.3, https://www.r-project.org/).

There was a significant overlap of various AS in PD patients. The prevalence of AS (at least one SCOPA-AUT domain with a score greater than or equal to 1) in PD was 97.20%. The proportion of the patients without AS or with a single autonomic dysfunction was only 12.32%, while up to 30.67% of the patients had three out of six coexisting AS. Patients with two out of six coexisting AS, three out of six coexisting AS, and four out of six coexisting AS accounted for 77.45% (Fig. 1A). Figure 1B showed details of the coexisting AS.

Characteristics of PD patients according to the motor subtypes (PIGD: n = 297, 41.60%; TD: n = 343, 48.04%; and indeterminate: n = 74, 10.36%) were shown in Table 2. PIGD was featured by the lower level of education, older age, longer disease duration, larger proportion of advanced disease stage, lower MDS-UPDRS part I score, higher MDS-UPDRS parts II and IV scores, higher LED, higher prevalence of depression than the other subtypes. In TD, proportion of early disease stage, scores of MDS-UPDRS part III and MOCA, and prevalence of pRBD were higher, contrast to the lower MDS-UPDRS part II score, LED, and prevalence of depression than PIGD and indeterminate.

**Table 2:** PD motor subtypes: demographic and clinical characteristics.

	Indeterminate (n = 74)	PIGD (n = 297)	TD (n = 343)	*p* Value
Age, years (median [IQR])	62.71 [56.22, 68.75]*	67.00 [62.00, 71.00]	63.18 [56.80, 69.02]*	< 0.001
Gender female, n (%)	29 (39.18)	125 (42.09)	122 (35.56)	0.239
Education, years (median [IQR])	14.00 [10.25, 16.00] *	11.00 [9.00, 14.00]	16.00 [13.00, 18.00] *	< 0.001
Duration, years (median [IQR])	0.57 [0.26, 2.04] *	3.14 [0.75, 6.60]	0.40 [0.22, 1.01] *	< 0.001
Age of onset, years (median [IQR])	59.84 [53.03, 67.40]	62.00 [56.14, 67.00]	61.08 [54.85, 66.82]	0.252
Hoehn and Yahr stage, n (%)				< 0.001
Stage 1	21 (28.38)^a^	59 (19.87)	158 (46.06)*	
Stage 1.5	15 (20.27)^a^	66 (22.22)	12 (3.50)*	
Stage 2	36 (48.65)	107 (36.03)	172 (50.15)*	
Stage ≥ 2.5	2 (2.70)*	36 (21.89)	1 (0.30)*	
MDS-UPDRS I score (mean (SD))	4.73 (3.65)*	3.66 (3.44)	4.67 (3.82)*	0.001
MDS-UPDRS II score (mean (SD))	7.97 (4.16)*^a^	9.89 (5.27)	5.36 (3.85)*	< 0.001
MDS-UPDRS III score (mean (SD))	15.89 (8.32)	15.85 (7.15)	19.32 (9.74)*	< 0.001
MDS-UPDRS IV score (mean (SD))	0.30 (0.57)*	1.26 (1.74)	0.07 (0.30)*	< 0.001
LED, mg/day (mean (SD))	108.45 (179.77) *^a^	329.74 (282.84)	39.94 (117.67) *	< 0.001
MOCA score (median [IQR])	26.00 [25.00, 28.00]	25.00 [24.00, 25.00]	27.00 [25.00, 29.00]*	< 0.001
Depression, n (%)	36 (48.65)*	236 (79.46)	78 (22.74)*	< 0.001
EDS, n (%)	11 (14.86)	27 (9.09)	42 (12.24)	0.259
pRBD, n(%)	20 (27.03)	61 (20.54)	111 (32.36)*	0.003

Abbreviations: *PD* Parkinson’s disease, *PIGD* postural instability and gait disturbance, *TD* tremor dominant, *MDS-UPDRS* Movement Disorder Society revision of the Unified Parkinson’s Disease Rating Scale, *MOCA* Montreal Cognitive Assessment, *EDS* excessive daytime sleepiness, *pRBD* probable rapid eye movement sleep behavior disorder, *SCOPA-AUT* Scales for Outcomes in Parkinson’s Disease–Autonomic Dysfunction, *IQR* interquartile range, *SD* standard deviation.

*p < 0.05 compared with the PIGD subtype.

^a^*p* < 0.05 compared with the tremor-dominant subtype.

Table 3 showed the characteristics of PD patients within each domain autonomic dysfunction group. Patients with gastrointestinal symptom and urinary symptom were older and had higher onset age. A mild sex distribution showed male predominance in the sexual dysfunction symptom group. As for education, the sexual dysfunction symptom and pupillomotor symptom groups had higher level of education. Patients with gastrointestinal symptom or thermoregulatory symptom tended to have longer durations, higher LEDs, higher rates of depression, and lower MOCA scores, contrast to those with pupillomotor symptom or sexual dysfunction symptom. More patients with gastrointestinal symptom or sexual dysfunction symptom belonged to Hoehn & Yahr stage 1 and stage 2. On the whole, patients with AS were more likely to have higher MDS-UPDRS I, II, III and IV scores, whereas the patients with the sexual dysfunction symptom had lower MDS-UPDRS II and IV scores, respectively. With regard to the sleep disturbances in PD, gastrointestinal symptom, cardiovascular symptom, and sexual dysfunction symptom groups had higher rates of EDS. And patients with AS were more likely to suffer from pRBD, except for the pupillomotor symptom group.

**Table 3:** Patients with PD classified by autonomic symptoms.

	Gastrointestinal symptom			Urinary symptom			Cardiovascular symptom			Thermoregulatory symptom			Pupillomotor symptom			Sexual function symptom		
	No (n = 156)	Yes (n = 558)	*p* value	No (n = 58)	Yes (n = 656)	*p* value	No (n = 463)	Yes (n = 251)	*p* value	No (n = 423)	Yes (n = 291)	*p* value	No (n = 592)	Yes (n = 122)	*p* value	No (n = 517)	Yes (n = 197)	*p* value
Age, years	62.02 [55.33, 67.00]	66.00 [59.94, 70.04]	< 0.001	63.00 [58.99, 67.00]	65.00 [59.00, 70.00]	0.022	65.00 [59.00, 69.85]	65.95 [59.29, 70.68]	0.214	65.00 [59.00, 70.00]	65.00 [59.00, 70.00]	0.741	65.00 [59.37, 70.00]	64.48 [56.80, 70.25]	0.591	65.00 [59.00, 70.00]	64.32 [58.88, 69.43]	0.343
Gender female, n (%)	61 (39.10)	215 (38.5)	0.971	29 (50.0)	247 (37.7)	0.087	181 (39.1)	95 (37.8)	0.806	151 (35.7)	125 (43.0)	0.060	225 (38.0)	51 (41.8)	0.495	214 (41.4)	62 (31.5)	0.019
Education, years	14.00[11.00, 17.00]	14.00 [11.00, 16.00]	0.114	13.00 [10.00, 15.75]	14.00 [11.00, 16.00]	0.082	14.00 [11.00, 16.00]	13.00 [11.00, 16.00]	0.067	15.00 [11.00, 16.00]	12.00 [11.00, 16.00]	< 0.001	14.00 [11.00, 16.00]	14.00 [12.00, 16.00]	0.029	12.00 [11.00, 16.00]	16.00 [14.00, 18.00]	< 0.001
Duration, years	0.62 [0.26, 1.87]	0.87 [0.29, 3.50]	0.015	1.10 [0.45, 3.56]	0.76 [0.28, 3.05]	0.292	0.79 [0.29, 2.90]	0.78 [0.28, 3.49]	0.731	0.68 [0.26, 2.53]	0.97 [0.29, 4.68]	0.002	0.92 [0.30, 3.46]	0.50 [0.23, 1.41]	0.001	1.49 [0.33, 4.33]	0.40 [0.23, 0.78]	< 0.001
Age of onset, years	59.38 [52.95,64.01]	62.00 [56.00, 67.00]	0.001	59.00 [55.00, 63.90]	61.90 [55.33, 67.00]	0.040	61.00 [55.20, 66.87]	62.00 [54.94, 67.00]	0.464	61.77 [55.78, 67.00]	61.00 [54.49, 66.61]	0.303	61.46 [55.72, 66.66]	60.99 [53.96, 68.18]	0.989	61.00 [55.00, 66.62]	62.59 [56.29, 68.06]	0.061
Hoehn and Yahr stage, n (%)			< 0.001			0.077			0.282			0.013			0.077			< 0.001
Stage 1	77 (49.36)	161 (28.85)		25 (43.10)	213 (32.47)		162 (34.99)	76 (30.28)		148 (34.99)	90 (30.93)		198 (33.45)	40 (32.79)		154 (29.79)	84 (42.64)	
Stage 1.5	19 (12.18)	74 (13.27)		12 (20.69)	81 (12.35)		62 (13.39)	31 (12.35)		44 (10.40)	49 (16.84)		86 (14.53)	7 ( 5.74)		92 (17.79)	1 (0.51)	
Stage 2	52 (33.33)	263 (47.13)		18 (31.03)	297 (45.27)		201 (43.41)	114 (45.42)		199 (47.04)	116 (39.86)		253 (42.74)	62 (50.82)		206 (39.85)	109 (55.33)	
Stage ≥ 2.5	8 (5.13)	60 (10.75)		3(5.17)	65 (9.91)		38 (8.21)	30 (11.95)		32 (7.57)	46 (15.80)		55 (9.29)	13 (10.66)		65 (12.57)	3 (1.52)	
MOCA score	26.00 [25.00, 28.00]	25.00 [24.00, 28.00]	0.014	25.00 [24.00, 26.75]	25.00 [25.00, 28.00]	0.054	25.00 [24.50, 28.00]	25.00 [24.50, 28.00]	0.397	26.00 [25.00, 28.00]	25.00 [24.00, 27.00]	< 0.001	25.00 [24.00, 28.00]	27.00 [25.00, 29.00]	< 0.001	25.00 [24.00, 27.00]	27.00 [26.00, 29.00]	< 0.001
LED, mg/day	96.95 (173.39)	187.34 (263.84)	< 0.001	181.90 (209.29)	166.32 (252.99)	0.649	158.45 (241.30)	184.44 (263.97)	0.184	129.54 (209.41)	222.90 (290.20)	< 0.001	184.53 (254.47)	85.37 (206.64)	< 0.001	227.33 (266.28)	10.79 (76.77)	< 0.001
Depression, n (%)	60 (38.46)	290 (51.97)	0.004	36 (62.07)	314 (47.87)	0.053	208 (45.93)	142 (56.57)	0.004	178 (42.08)	172 (59.11)	< 0.001	307 (51.86)	43 (35.25)	0.001	317 (61.32)	33 (16.75)	< 0.001
EDS, n (%)	10 ( 6.41)	70 (12.54)	0.045	2 (3.45)	78 (11.89)	0.082	43 (9.29)	37 (14.74)	0.037	40 (9.46)	40 (13.75)	0.096	62 (10.47)	18 (14.75)	0.227	43 (0.08)	37 (19.28)	< 0.001
pRBD, n (%)	23 (14.74)	169 (30.28)	< 0.001	7 (12.07)	471 (71.80)	0.012	107 (23.11)	85 (33.86)	0.003	100 (23.64)	92 (31.62)	0.023	307 (51.86)	43 (35.25)	0.001	110 (21.28)	82 (42.62)	< 0.001
MDS-UPDRS I score	2.83 (2.53)	4.66 (3.85)	< 0.001	2.05 (1.99)	4.45 (3.73)	< 0.001	3.74 (3.09)	5.22 (4.42)	< 0.001	3.74 (3.18)	5.00 (4.20)	< 0.001	3.78 (3.24)	6.57 (4.67)	< 0.001	3.51 (3.17)	6.22 (4.17)	< 0.001
MDS-UPDRS II score	4.70 (3.75)	8.30 (5.03)	< 0.001	4.86 (4.14)	7.75 (5.01)	< 0.001	6.69 (4.38)	9.02 (5.69)	< 0.001	6.49 (4.52)	8.99 (5.29)	< 0.001	7.38 (4.86)	8.16 (5.61)	0.118	7.88 (5.14)	6.55 (4.50)	0.002
MDS-UPDRS III score	15.72 (8.04)	18.03 (8.90)	0.004	12.71 (8.53)	17.95 (8.66)	< 0.001	17.04 (8.88)	18.40 (8.51)	0.048	17.87 (9.12)	17.01 (8.21)	0.200	17.05 (8.75)	19.82 (8.52)	0.001	16.26 (8.50)	20.82 (8.61)	< 0.001
MDS-UPDRS IV score	0.32 (0.80)	0.66 (1.38)	0.004	0.55 (0.86)	0.59 (1.32)	0.835	0.48 (1.04)	0.78 (1.63)	0.003	0.35 (0.89)	0.92 (1.65)	< 0.001	0.59 (1.19)	0.54 (1.69)	0.675	0.77 (1.33)	0.09 (1.01)	< 0.001
Subtypes, n (%)			0.045			0.585			0.044			< 0.001			0.003			< 0.001
Indeterminate	16 (10.26)	58 (10.39)		8 (13.80)	66 (10.06)		39 (8.42)	35 (13.94)		33 ( 7.81)	41 (14.09)		51 (8.61)	23 (18.86)		48 (9.28)	26 (13.20)	
PIGD	52 (33.33)	245 (43.91)		25 (43.10)	272 (41.46)		191 (41.25)	106 (42.23)		135 (31.91)	162 (55.67)		251 (42.40)	46 (37.70)		261 (50.48)	36 (18.27)	
TD	88 (56.41)	255 (45.70)		25 (43.10)	318 (48.48)		233 (50.33)	110 (43.83)		255 (60.28)	88 (30.24)		290 (48.99)	53 (43.44)		208 (40.24)	135 (68.53)	

Abbreviations: *PD* Parkinson’s disease, *MOCA* Montreal Cognitive Assessment. All values expressed as mean (standard deviation), frequency (%), or median [interquartile range], *MDS-UPDRS* Movement Disorder Society revision of the Unified Parkinson’s Disease Rating Scale, *LED* levodopa equivalent dose, *EDS* excessive daytime sleepiness, *pRBD* probable rapid eye movement sleep behavior disorder, *PIGD* postural instability and gait disturbance, *TD* tremor dominant. All values expressed as mean (standard deviation), frequency (%), or median [interquartile range].

Final logistic regression analyses of each domain autonomic dysfunction were showed in Table 4. Age, Hoehn and Yahr stage, MDS-UPDRS I and II scores, and pRBD were predictors of gastrointestinal symptom. Age, MDS-UPDRS I and II scores, and depression were significant factors of urinary symptom. MDS-UPDRS I and II scores, pRBD and the PIGD subtype were predictors of cardiovascular symptom. MDS-UPDRS I, III and IV scores, and the TD subtype helped to predict thermoregulatory symptom. MDS-UPDRS I and IV scores, depression, and the TD subtype were important variables of pupillomotor symptom. And age of onset, Hoehn and Yahr stage, MDS-UPDRS I score, depression, LED, and the PIGD subtype exerted an important effect on sexual symptom. No collinearity existed among variables. GBDT identified MDS-UPDRS I, II and III scores, age, duration, and age of onset as top 6 predictors of gastrointestinal symptom. MDS-UPDRS I, II and III scores, duration, age of onset, and age were selected as top 6 predictors of urinary symptom. MDS-UPDRS I, II and IV scores, depression, age of onset, age and motor subtype were identified as top 6 predictors of cardiovascular symptom. Motor subtype, MDS-UPDRS I and II scores, education, duration, and LED were derived to help to predict thermoregulatory symptom. MDS-UPDRS I and III scores, duration, motor subtype, and age were the top 6 predictors of pupillomotor symptom in GBDT model. And GBDT recognized LED, MDS-UPDRS I score, education, age, age of onset, and motor subtype as the top 6 predictors of sexual symptom.

**Table 4:** Logistic regression analyses of autonomic symptoms of patients with PD.

	OR	95% CI	*p* value
Gastrointestinal symptom			
Age	1.04	1.02 to 1.06	0.001
Hoehn and Yahr stage			
Stage 1	Ref		
Stage 1.5	0.73	0.35 to 1.52	0.39
Stage 2	1.35	0.85 to 2.13	0.20
Stage 2.5	3.95	0.70 to 74.9	0.20
Stage 3	0.14	0.04 to 0.48	0.002
MDS-UPDRS I score	1.20	1.10 to 1.31	< 0.001
MDS-UPDRS II score	1.16	1.09 to 1.24	< 0.001
pRBD			
No	Ref		
Yes	2.13	1.26–3.70	0.006
Urinary symptom			
Age	1.05	1.01–1.08	0.006
MDS-UPDRS I score	1.30	1.14–1.52	0.0003
MDS-UPDRS II score	1.17	1.08–1.28	0.0004
Depression			
No	Ref		
Yes	0.36	0.18–0.71	0.0003
Cardiovascular symptom			
MDS-UPDRS I score	1.11	1.05–1.17	0.0002
MDS-UPDRS II score	1.07	1.02–1.11	0.002
pRBD			
No	Ref		
Yes	1.45	0.99–2.13	0.056
Subtype			
PIGD	Ref		
TD	1.64	1.05–2.58	0.03
Indeterminate	2.27	1.29–4.02	0.004
Thermoregulatory symptom			
MDS-UPDRS I score	1.16	1.09–1.22	< 0.001
MDS-UPDRS III score	0.97	0.95–0.99	0.01
MDS-UPDRS IV score	1.32	1.12–1.57	0.001
Subtype			
TD	Ref		
PIGD	2.70	1.77–4.13	< 0.001
Indeterminate	3.12	1.80–5.44	< 0.001
Pupillomotor symptom			
MDS-UPDRS I score	1.18	1.12–1.25	< 0.001
MDS-UPDRS IV score	1.33	1.09–1.65	0.006
Depression			
No	Ref		
Yes	0.47	0.25–0.85	0.01
Subtype			
TD	Ref		
PIGD	1.86	1.08–3.19	0.03
Indeterminate	3.35	1.79–6.23	< 0.001
Sexual function symptom			
Age of onset	1.03	1.01–1.05	0.003
Hoehn and Yahr stage			
Stage 1	Ref		
Stage 1.5	0.10	0.005–0.56	0.03
Stage 2	0.83	0.55–1.25	0.38
Stage 2.5	0.79	0.04–5.43	0.83
Stage 3	1.12	0.04–0.48	0.91
MDS-UPDRS I score	1.14	1.09–1.21	< 0.001
Depression			
No	Ref		
Yes	0.45	0.25–0.80	0.008
LED	0.99	0.990–0.996	< 0.001
Subtype			
Indeterminate	Ref		
PIGD	0.45	0.22–0.94	0.03
TD	0.68	0.36–1.27	0.23

Abbreviations: *PD* Parkinson’s disease, *Ref* reference, *OR* odds ratio, *CI* confidence interval, *MDS-UPDRS* Movement Disorder Society revision of the Unified Parkinson’s Disease Rating Scale, *pRBD* probable rapid eye movement sleep behavior disorder, *PIGD* postural instability and gait disturbance, *TD* tremor dominant, *LED* levodopa equivalent dose, *PIGD* postural instability and gait disturbance, *TD* tremor dominant.

**Figure 2:**
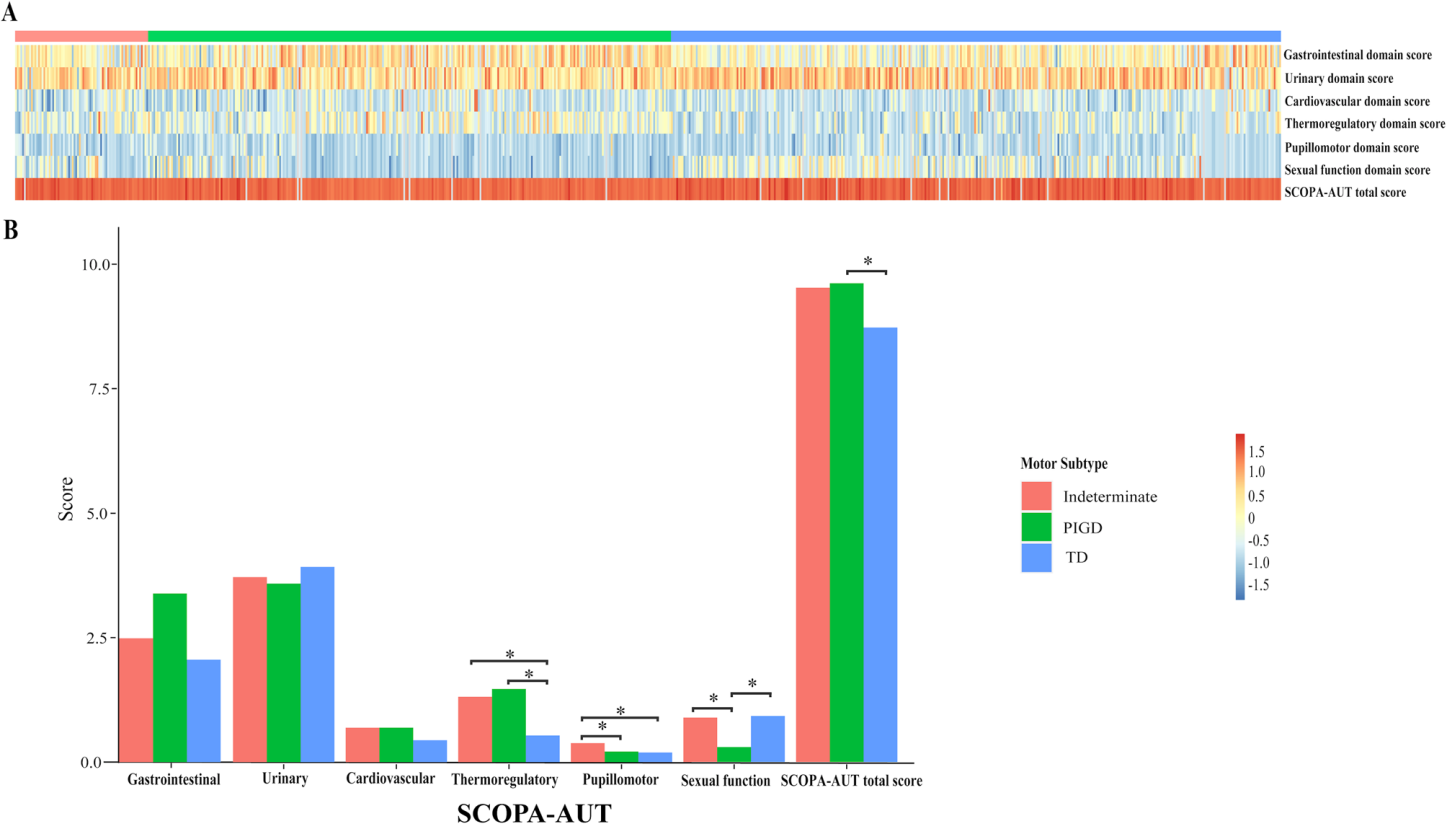
(**A**) Heat-maps of gastrointestinal, urinary, cardiovascular, and sexual dysfunction domain SCOPA-AUT scores for indeterminate, TD, and PIGD groups in PD. Data were log2-transformated with normalization and red implied increased expression while blue implied decreased expression. (**B**) Clinical motor subtypes and autonomic symptoms. *p ≤ 0.05. PD, Parkinson’s disease; TD, tremor dominant; PIGD; postural instability and gait disturbances; SCOPA-AUT, Scales for Outcomes in Parkinson's Disease-Autonomic dysfunction. All figures were created with the use of R software (version 3.3.3, https://www.r-project.org/).

Figure 2A showed the heat-maps of total SCOPA-AUT score and 6 domain scores among TD, PIGD and indeterminate. After adjusting for age, and MDS-UPDRS I and II scores, the gastrointestinal domain and urinary domain scores did not significantly differ among the groups (Fig. 2B). After adjusting for MDS-UPDRS I and II scores, the cardiovascular domain score did not significantly differ among the groups (Fig. 2B). After adjusting for MDS-UPDRS I score, TD had a lower thermoregulatory domain score than PIGD (*p* < 0.001) and indeterminate (*p* < 0.001, Fig. 2B). After adjusting for MDS-UPDRS I score, indeterminate had a higher pupillomotor domain score than PIGD (*p* = 0.05) and TD (*p* = 0.004, Fig. 2B). After adjusting for MDS-UPDRS I score, age of onset, and LED, PIGD had a lower sexual dysfunction domain score than TD (*p* = 0.02) and indeterminate (*p* = 0.05) groups (Fig. 2B). After adjusting for age, and MDS-UPDRS I and II scores, PIGD had a higher SCOPA-AUT total score than TD (*p* = 0.01, Fig. 2B).

In addition, correlation between total SCOPA-AUT score and PD severity (according to the Hoehn and Yahr stage) was significant (r = 0.202; *p* < 0.001). Exploratory comparison of mean total SCOPA-AUT performance in patients with different Hoehn and Yahr stages showed that there was a significant increase in the score as PD progressed (*p* < 0.001).

## Discussion

In this cross-sectional study, we systematically assessed the metric properties of AS in a large PD sample from multiple centers. The highly complex relationship between AS and PD has already been studied for decades. Notably, this was the first study to identify significant links between AS and PD motor subtypes.

### Extensive autonomic dysfunction performances in PD

SCOPA-AUT data in the current study showed a good reliability profile, with no obvious test floor or ceiling effect. The mean total SCOPA-AUT score in PD group was higher than controls, confirming that PD patients experienced significant autonomic dysfunction. This mean score was lower when comparing to previous studies^31,32^. It might be due to the absence of the very advanced PD patients since correlation was found between the severity of PD and total SCOPA-AUT score (r = 0.202). This finding was in agreement with previous studies showing that mean total SCOPA-AUT score tended to be higher as PD severity increased^33,34^.

PD patients reported dysfunction in all domains of SCOPA-AUT, with mean scores ranging from 3.75 to 0.19. In addition, we found a significant overlap in domain autonomic dysfunction. The prevalence of patients without AS or with a single autonomic dysfunction was very low (Fig. 1). The most common AS emerged in urinary and gastrointestinal domains. And the most prominent difference between two groups was found in gastrointestinal domain (2.24 versus 0.68). The peripheral ANS can be divided into sympathetic (cholinergic and noradrenergic), parasympathetic pathways, and the enteric nervous system (ENS) anatomically, and dysfunction of any particular component would lead to characteristic symptoms. Parasympathetic cholinergic impairment would bring about urinary retention, hyposialorrhea, an invariable pulse rate, and erectile failure, while sympathetic cholinergic impairment would bring about decreased sweating. Sympathetic noradrenergic dysfunction would be responsible for orthostatic hypotension and orthostatic intolerance. And ENS failure would present as delayed gastric emptying and constipation^5^. Hence, our results showed that autonomic dysfunction in PD existed across a broad spectrum of severity with a variety of components of ANS as important clinical entities^35^.

### Heterogeneous pattern of autonomic dysfunction in PD

In the current research, contributing predictors of the single domain autonomic symptom were firstly discovered. Based on @@, gastrointestinal symptom was closely linked with old age, advanced disease stage, pRBD, and high MDS-UPDRS I and II scores. Old age, advanced disease stage, depression, and high MDS-UPDRS I and II scores played an important role in urinary symptom. In addition, TD and PIGD suffering from pRBD with higher MDS-UPDRS I and II scores tended to have cardiovascular problem. TD and PIGD who scored high in MDS-UPDRS I, III, and IV scores might be more likely to suffer from thermoregulatory symptom. Moreover, TD and PIGD, high MDS-UPDRS I and IV scores, and depression might result in pupillomotor symptom. As for sexual symptom, PD onset age, disease stage, LED, the incidence of depression, MDS-UPDRS I score, and motor subtypes might be its predictors accordingly.

It was of worth that the results derived from logistic models and GBDT models were similar. From a methodological point of view, these two models were fundamentally different, which hinted to the robustness of the results, highlighting the hypothesis that pathologic involvement in PD was not only of brain dopaminergic systems but also of non-dopaminergic systems in the brain as well as peripheral and autonomic systems. The patchy and heterogeneous AS observed here implied that every single component of ANS was involved by the Lewy pathology of PD^8–10,36–39^. Furthermore, according to our study, these widespread pathologies throughout the autonomic networks tended to follow an erratic rather than a stepwise progression. Thus, the assessment of the density of the autonomic neurons in the different components of ANS would be an important step toward verifying the speculation of complex pathophysiology of dysautonomia in PD.

### Links between motor subtypes and autonomic function in PD

Heterogeneity was also observed in PD subtypes. Compared with the non-PIGD, PIGD tended to be older and to have more advanced stage, longer disease duration, and more severe motor complications. In addition, more mood impairment such as depression and higher doses of levodopa therapy were associated with PIGD. Differences were also observed in cognitive performance and pRBD among different subtypes. Cognitive deficits seemed to be less severe, while pRBD were more frequent in TD as compared with non-TD.

It was notable that we found distinctions in autonomic function within TD, PIGD and indeterminate, which could be helpful in individuating patients at risk of variable AS to initiate possible pharmacological and non-pharmacological interventions in clinical trials. PIGD and indeterminate were the predominant clinical motor subtypes in the pupillomotor and thermoregulatory symptoms as compared with TD. Correspondingly, TD showed significantly lower pupillomotor and thermoregulatory domain scores after correcting for confounding factors (Fig. 2B). TD and indeterminate were more likely to suffer from cardiovascular problem as compared with PIGD, although the cardiovascular domain score did not significantly differ among these three subtypes (Fig. 2B). The odd in sexual dysfunction symptom was significant for PIGD as compared with indeterminate subtype. In response, PIGD had a lower score in the sexual dysfunction domain (Fig. 2B). Gastrointestinal and urinary symptoms seemed not to be associated with a specific motor subtype (Table 4 and Fig. 2B), which might be the reason for their high incidences in PD.

Importantly, the significant relation between subtypes and autonomic dysfunction discovered here has never been reported. With learning more about PD subtypes, it is becoming abundantly clear that proper subtype-specific markers might provide insights into mechanisms and improve therapeutic and epidemiologic clinical study designs in PD. Our findings implied that these AS might occur in association with the degree and distribution of the degeneration of brain dopaminergic/non-dopaminergic neurons, which could determine certain motor and non-motor phenotypes of PD, which had direct clinical relevance with the potential to be new subtype-specific markers. In addition, these valuable findings also suggested that dysautonomia might interact with motor state, adding evidence for the assumption that the initial site of disease in PD might not necessarily be in the substantia nigra but might be in the nuclei of the caudal brainstem or even in the peripheral organs and peripheral nervous system^40^.

### Limitations

One limitation was the lack of ANS histological evaluations. Comorbidities such prostatic hyperplasia might influence SCOPA-AUT items and could bias some results. The principal reason was that some of them were considered to be too invasive. Other limitations were the difference in the distribution of PD subtype in different literature and the small sample size of advanced PD patients, hindering further stratification of patients into sub-groups with different motor severity. And the impact of symptomatic medications such as hypotension caused by levodopa remained unknown, which needed to be promoted in larger and longitudinal studies. Finally, as one could argue that because classification of subtype was done at baseline, this classification could anyway change dynamically and there might be a transition and resulting influence on AS during the course, we tried to control for disease duration. And the transition and resulting influence on AS needed to be addressed in future analyses. Moreover, our preliminary approach to correlating AS with PD subtypes of a large sample, which was close to ‘a real-world clinical setting’, was potentially valuable. Three subtypes of PD differed in autonomic performance, further longitudinal studies with objective assessments of autonomic function would be necessary and shed more light on these differential findings.

## Conclusions

In conclusion, this study showed that PD is heterogeneous, both in terms of AS and motor subtypes. Significant associations of AS with motor subtypes identified here suggested the contribution of peripheral and autonomic systems change in etiological models of PD, as well as the multi-level interaction between NMS and motor state, reinforcing and extending the understanding of mechanisms underlying AS and PD motor subtypes.

### Supplementary Information

Supplementary Information 1.

Supplementary Information 2.

## Data Availability

The datasets generated during and/or analysed during the current study are available from the corresponding author on reasonable request.

## References

[1] Armstrong MJ, Okun MS (2020). Diagnosis and treatment of parkinson disease: A review. JAMA.

[2] Schapira AHV, Chaudhuri KR, Jenner P (2017). Non-motor features of Parkinson disease. Nat. Rev. Neurosci..

[3] Chaudhuri KR, Prieto-Jurcynska C, Naidu Y, Mitra T, Frades-Payo B, Tluk S (2010). The nondeclaration of nonmotor symptoms of Parkinson’s disease to health care professionals: An international study using the nonmotor symptoms questionnaire. Mov. Disord..

[4] Jain S (2010). Multi-organ autonomic dysfunction in Parkinson disease. Parkinsonism Relat. Disord..

[5] McCorry LK (2007). Physiology of the autonomic nervous system. Am. J. Pharm. Educ..

[6] Kaufmann H, Goldstein DS (2013). Autonomic dysfunction in Parkinson disease. Handb. Clin. Neurol..

[7] Selikhova M, Williams DR, Kempster PA, Holton JL, Revesz T, Lees AJ (2009). A clinico-pathological study of subtypes in Parkinson’s disease. Brain.

[8] Wojtala J, Heber A, Neuser P, Heller J, Kalbe E, Rehberg SP (2019). Cognitive decline in Parkinson's disease: The impact of the motor phenotype on cognition. J. Neurol. Neurosurg. Psychiatry..

[9] Suzuki K, Okuma Y, Uchiyama T, Miyamoto M, Sakakibara R, Shimo Y (2017). Impact of sleep-related symptoms on clinical motor subtypes and disability in Parkinson's disease: A multicentre cross-sectional study. J. Neurol. Neurosurg. Psychiatry..

[10] Burn DJ, Landau S, Hindle JV, Samuel M, Wilson KC, Hurt CS (2012). Parkinson's disease motor subtypes and mood. Mov. Disord..

[11] Del Tredici K, Braak H (2012). Spinal cord lesions in sporadic Parkinson’s disease. Acta. Neuropathol..

[12] Beach TG, Adler CH, Serrano G, Sue LI, Walker DG, Dugger BN (2016). Prevalence of submandibular gland synucleinopathy in parkinson’s disease, dementia with Lewy bodies and other Lewy body disorders. J. Parkinsons. Dis..

[13] Leclair-Visonneau L, Magy L, Volteau C, Clairembault T, Le Dily S, Préterre C (2018). Heterogeneous pattern of autonomic dysfunction in Parkinson's disease. J. Neurol..

[14] Verbaan D, Marinus J, Visser M, van Rooden SM, Stiggelbout AM, van Hilten JJ (2007). Patient-reported autonomic symptoms in Parkinson disease. Neurology.

[15] Jost WH (2003). Autonomic dysfunctions in Parkinson’s disease. J. Neurol..

[16] Gu SC, Ye Q, Wang CD, Zhao SR, Zhou J, Gao C (2022). Pingchan granule for motor symptoms and non-motor symptoms of Parkinson's disease: A randomized, double-blind, Placebo-controlled study. Front. Pharmacol..

[17] Parkinson Progression Marker Initiative (2011). The Parkinson progression marker initiative (PPMI). Prog. Neurobiol..

[18] Postuma RB, Berg D, Stern M, Poewe W, Olanow CW, Oertel W (2015). MDS clinical diagnostic criteria for Parkinson's disease. Mov. Disord..

[19] Hoehn MM, Yahr MD (2011). Parkinsonism: Onset, progression and mortality. 1967. Neurology.

[20] Hoops S, Nazem S, Siderowf AD, Duda JE, Xie SX, Stern MB (2009). Validity of the MoCA and MMSE in the detection of MCI and dementia in Parkinson disease. Neurology.

[21] Kashihara K, Kondo T, Mizuno Y (2014). Official Japanese version of the movement disorder society-unified Parkinson’s disease rating scale: Validation against the original English version. Mov. Disord. Clin. Pract..

[22] Stebbins GT, Goetz CG, Burn DJ, Jankovic J, Khoo TK, Tilley BC (2013). How to identify tremor dominant and postural instability/gait difficulty groups with the movement disorder society unified Parkinson's disease rating scale: Comparison with the unified Parkinson's disease rating scale. Mov. Disord..

[23] Matsushima M, Yabe I, Hirotani M, Kano T, Sasaki H (2014). Reliability of the Japanese version of the scales for outcomes in Parkinson’s disease-autonomic questionnaire. Clin. Neurol. Neurosurg..

[24] Johns MW (1991). A new method for measuring daytime sleepiness: The Epworth sleepiness scale. Sleep.

[25] Miyamoto T, Miyamoto M, Iwanami M, Ko-bayashi M, Nakamura M, Inoue Y (2009). The REM sleep behavior disorder screening questionnaire: validation study of a Japanese version. Sleep. Med..

[26] Weintraub D, Saboe K, Stern MB (2007). Effect of age on geriatric depression scale performance in Parkinson's disease. Mov. Disord..

[27] Tomlinson CL, Stowe R, Patel S, Rick C, Gray R, Clarke CE (2010). Systematic review of levodopa dose equivalency reporting in Parkinson's disease. Mov. Disord..

[28] Austin PC, Jembere N, Chiu M (2018). Propensity score matching and complex surveys. Stat. Methods. Med. Res..

[29] Kim H (2019). Propensity score analysis in non-randomized experimental designs: An overview and a tutorial using R software. New. Dir. Child. Adolesc..

[30] Friedman JH (2001). Greedy function approximation: A gradient boosting machine. Ann Stat..

[31] Kim JY, Song IU, Koh SB, Ahn TB, Kim SJ, Cheon SM (2017). Validation of the Korean version of the scale for outcomes in Parkinson's disease-autonomic. J. Mov. Disord..

[32] Bostantjopoulou S, Katsarou Z, Danglis I, Karakasis H, Milioni D, Falup-Pecurariu C (2016). Self-reported autonomic symptoms in Parkinson's disease: Properties of the SCOPA-AUT scale. Hippokratia.

[33] Verbaan D, Marinus J, Visser M, van Rooden SM, Stiggelbout AM, van Hilten JJ (2007). Patient–reported autonomic symptoms in Parkinson disease. Neurology.

[34] Rodriguez-Blazquez C, Forjaz MJ, Frades-Payo B, de Pedro-Cuesta J, Martinez-Martin P, Longitudinal Parkinson’s Disease Patient Study, Estudio Longitudinal de Pacients con Enfermedad da Parkinson Group (2010). Independent validation of the scales for outcomes in Parkinson’s diseases–autonomic (SCOPA–AUT). Eur. J. Neurol..

[35] Micieli G, Tosi P, Marcheselli S, Cavallini A (2003). Autonomic dysfunction in Parkinson’s disease. Neurol. Sci..

[36] Gelpi E, Navarro-Otano J, Tolosa E (2014). Multiple organ involvement by alpha-synuclein pathology in Lewy body disorders. Mov. Disord..

[37] Beach TG, Adler CH, Sue LI, Vedders L, Lue L, Akiyama H (2010). Multi-organ distribution of phosphorylated alpha-synuclein histopathology in subjects with Lewy body disorders. Acta. Neuropathol..

[38] van Dijk JG, Haan J, Zwinderman K, Kremer B, van Hilten BJ, Roos RA (1993). Autonomic nervous system dysfunction in Parkinson’s disease: Relationships with age, medication, duration, and severity. J. Neurol. Neurosurg. Psychiatry..

[39] Jellinger KA (2012). Neuropathology of sporadic Parkinson’s disease: Evaluation and changes of concepts. Mov. Disord..

[40] Orimo S, Ghebremedhin E, Gelpi E (2018). Peripheral and central autonomic nervous system: Does the sympathetic or parasympathetic nervous system bear the brunt of the pathology during the course of sporadic PD?. Cell. Tissue. Res..

